# Toll-like receptors 2, 4 and 7, interferon-gamma and interleukin 10, and programmed death ligand 1 transcripts in skin from dogs of different clinical stages of leishmaniosis

**DOI:** 10.1186/s13071-019-3827-7

**Published:** 2019-12-05

**Authors:** Laura Ordeix, Sara Montserrat-Sangrà, Pamela Martínez-Orellana, Marta Baxarias, Laia Solano-Gallego

**Affiliations:** 1 Department de Medicina i Cirurgia Animals, Facultat de Veterinària, Universitat Autònoma de Barcelona, Bellaterra, Spain; 2 Hospital Clínic Veterinari, Universitat Autònoma de Barcelona, Bellaterra, Spain

**Keywords:** Leishmaniosis, Canine, *Leishmania infantum*, Skin, Cytokine, Toll-like receptors, Gene expression

## Abstract

**Background:**

Canine leishmaniosis (CanL) caused by *Leishmania infantum* can have several dermatological manifestations. The type of immune response elicited against the parasite appears to be at the basis for such clinical variability. Much of the work in CanL has focused on adaptive immune response and there are scarce data on the importance of the innate immune responses. Moreover, few studies have evaluated the immunological response in the cutaneous lesions in dogs naturally infected with *L. infantum* and with different degrees of disease severity, and no study has compared clinically-lesioned with normal-looking skin.

**Methods:**

We determined and compared the transcription of toll like receptors (TLRs) 2, 4 and 7, interferon gamma (IFN-γ), interleukin (IL) 10 and programmed cell death protein ligand (PD-L) 1 by real-time PCR in paired clinically-lesioned and normal-looking skin from 25 diseased dogs (mild disease-stage I (*n* = 11) and moderate to severe disease-stages II and III (*n* = 14) as well as in normal-looking skin from healthy dogs (*n* = 10) from a non-endemic area. We also assessed the association between the transcripts in clinically-lesioned and normal-looking skin of dogs with leishmaniosis with clinicopathological, immunological and parasitological findings.

**Results:**

Clinically-lesioned skin from mildly affected dogs was characterized by a significant upregulation of TLR2 (*P* < 0.0001) and IL-10 (*P* = 0.021) and downregulation of TLR7 (*P* = 0.004) when compared with more severely affected dogs. Normal-looking skin of mildly affected dogs was characterized by a significant lower expression of TLR7 (*P* = 0.003), IFN-γ (*P* < 0.0001) and PD-L1 (*P* = 0.001) when compared with more severely affected dogs. TLR2, TLR4, IL-10 and IFN-γ upregulation in clinically-lesioned skin was correlated with lower disease severity while TLR7 upregulation was correlated with markers of disease severity. Upregulation of TLR7, IL-10, IFN-γ and PD-L1 in normal-looking skin was correlated with disease severity.

**Conclusions:**

This study demonstrated different expression profiles of immune genes in clinically-lesioned and normal-looking skin among mildly and more severely affected dogs. These immunological conditions might favor the maintenance and replication of the parasite in the skin of more severely affected dogs.

## Background

Canine leishmaniosis (CanL) caused by *Leishmania infantum* is a zoonotic and an endemic disease in the Mediterranean basin among other areas such as south America, Middle East and Asia [1]. The complex immune response against the parasite is crucial for determining the outcome of infection [2]. In fact, subclinical infection is the result of an effective T helper 1 (Th1) cellular immunity, with the activation of macrophages by interferon-gamma (IFN-γ) and tumor necrosis factor-alpha (TNF-α) and the elimination of intracellular amastigotes *via* the l-arginine nitric oxide pathway [2, 3]. On the other hand, disease development and progression are often correlated with increased parasite burdens together with a strong but non-protective humoral immune response and reduced or absent T cell-mediated immunity [1].

Canine leishmaniosis is a systemic disease with varied clinical signs that range from a self-limiting disease to severe illness or even death [1]. Therefore, a clinical staging system for CanL that classifies the disease into four stages (stage I or mild disease, stage II or moderate disease, stage III or severe disease and stage IV or very severe disease) based on clinical signs, clinicopathological abnormalities, and measurement of anti-leishmanial antibodies was previously proposed [1] and recently updated [4].

Among the different clinical manifestations of CanL, dermatological disease is the most frequent [5, 6]. Cutaneous lesions are very pleomorphic from a clinical and histopathological point of view [5] and this clinicopathological variation might reflect a different host-parasite relationship and immune interactions [6, 7]. This is the particular case of papular dermatitis [6]. Papular dermatitis is a typical dermatological manifestation of CanL in an endemic area [5], which is classified as a stage I or mild disease in the absence of other clinicopathological abnormalities [4]. It has been suggested that there is strong T-cell mediated immunity against *L. infantum* that configures protection in these dogs [7–10]. On the other hand, other dermatological signs observed in CanL, such as exfoliative dermatitis, ulcerative dermatitis, onychogryphosis and muco-cutaneous nodular dermatitis are commonly observed in dogs with moderate to severe leishmaniosis [5, 6].

The immune response in CanL has been the focus of many investigations during the last years. However, much of this work focused on adaptive immune response and the data on the importance of the innate immune responses are scarce [11]. It is currently accepted that the immune response to the parasite is compartmentalized and different among organs [12, 13]. While the skin plays a major role in CanL immunopathogenesis, very limited data are available regarding normal-looking or lesioned skin from infected or diseased dogs [11].

A mixed Th1/Th2 cytokine profile in the dermis of dogs naturally infected with *L. infantum* has been described [14–17]. Recently, there has been a great interest in the involvement of Toll Like Receptors (TLRs) in the immunopathogenesis of CanL [16, 18–20]. TLRs are one of the most important pattern recognition receptor (PRR) molecules which recognize molecular structures characteristic of microbial pathogens and induce an inflammatory response [21]. Studies aimed to determine the role of TLRs in CanL are mainly *in vitro* studies performed on canine macrophages [22] or studies performed in blood [23], liver [16], spleen [16, 20, 24], intestine [19], brain [20, 24] or lymph node samples [16, 20]. TLR2 is one of the TLRs associated with the pathogenesis of cutaneous lesions in CanL [17, 25].

As discussed above, the suppression of cellular immunity is the most important aspect in the pathogenesis and progression of CanL [26]. During the last years, several studies have focused on the regulatory mechanisms and have demonstrated that programmed cell death protein (PD)-1 and its ligand (PD-L1) present in regulatory IgD^hi^ B cells are involved in the induction of T lymphocyte apoptosis *via* IL-10 production [27]. These studies have determined an increased PD1/PD-L1 expression in peripheral mononuclear cells as well as an increase in the expression of PD-L1 in splenic macrophages in dogs with leishmaniosis [27–29]. However, to the best of our knowledge, PD-L1 expression in the skin of diseased or infected dogs has not been investigated.

Only few published studies [14–17] have investigated the immunological response in the skin in dogs naturally infected with *L. infantum* and with different degrees of disease severity. Moreover, these studies have been mainly performed on normal-looking skin [15–17]. Therefore, the main objective of this study was to determine and compare the transcription of TLR2, TLR4, TLR7, IFN-γ, IL-10 and PD-L1 in paired clinically-lesioned and normal-looking skin from dogs with different clinical stages of leishmaniosis. Furthermore, we assessed the association between the transcripts in clinically-lesioned and normal-looking skin of dogs with leishmaniosis with clinicopathological, immunological and parasitological findings

## Methods

### Study groups

Twenty-five dogs with CanL and dermatological manifestation were prospectively selected from different veterinary centers in Catalonia and the Balearic Islands (Spain). These dogs were previously described in a published study aimed to characterise and compare the inflammatory pattern and the parasite burden in paired clinically-lesional and normal-appearing skin from the same dogs with dermatological manifestation due to CanL at different stages of disease [7]. Briefly, diagnosis was based on the observation of *L. infantum* on cytological and/or dermatopathological examination with or without *Leishmania-*specific immunohistochemical examination of cutaneous lesions [7]. Moreover, a complete blood count using the System Siemens Advia 120 hematology analyzer (Siemens Healthcare GmbH, Erlangen, Germany), a biochemical profile with the use of an Olympus AU 400 analyzer (CLIAwaived, San Diego, USA), serum protein electrophoresis using Hydrasys® (Sebia Electrophoresis, Norcross, USA), urinalysis with urinary protein/creatinine ratio calculation and quantitative serology for the detection of *L. infantum-*specific antibodies by means of a serial dilution in-house ELISA using the whole *L. infantum* antigens (strain: MHOM/FR/78/LEM75 zymodeme MON-1) were performed [30]. Blood *Leishmania* kinetoplast quantitative polymerase chain reaction (qPCR) was also performed [30]. Based on clinicopathological data, dogs were classified into three clinical stages: LeishVet stage I-mild disease characterized by persistent papular dermatitis (*n* = 11); II-moderate disease (*n* = 12); III-severe disease (*n* = 2) as previously reported [1]. However, for comparative analysis dogs were divided into two groups: Group A (11 dogs with LeishVet stage I and papular dermatitis); Group B (14 dogs with LeishVet stages II and III and exfoliative or ulcerative dermatitis). Normal-looking skin samples from 10 clinically healthy non-infected Beagle dogs from a non-endemic area (UK) (Group C) were used as control dogs.

### Skin biopsies

For all patients two skin fragments ≤ 0.5 cm from clinically lesioned skin and skin with normal appearance were collected. Normal-looking skin was obtained whenever possible from the lateral aspect of the neck. In cases where this region was affected, the biopsy was obtained from an area as far as possible from the macroscopically affected lesions. Each skin sample was then immediately cut into two halves. One half was fixed in 10% formalin for descriptive histopathology and analysis of the dermal parasite density as described previously [7] and the other one was submerged in RNA later (RNAlater^®^ Stabilization Solution, Ambion, Inc., Austin, USA), stored at 4 °C overnight and then keep at − 80 °C until used.

### RNA extraction

Before RNA isolation protocol, skin samples were thawed on ice and placed in lysis solution (TRI Reagent, RiboPure™ Kit, Ambion, Austin, USA) and homogenized with a rotor-stator homogenizer (T 10 basic ULTRA-TURRAX 230V IKA 3420000) using standard procedures. Total RNA was then isolated using the RiboPure™ Kit (Ambion) under strict RNase-free condition according to the manufacturer’s protocol. In order to remove contaminating DNA, a DNase digestion step was included using TURBO DNA-free™ DNase Treatment and Removal Reagents (Ambion) following the manufacturer’s instructions. RNA concentration was determined by a Nanodrop device (Thermo Fisher Scientific, Waltham, USA) and RNA integrity and quality were assessed by using an Agilent 2100 Bioanalyzer (Agilent Technologies, Santa Clara, USA) in some biopsies. Samples had a final concentration of 9.4–881.2 ng/µl. The majority of samples included in this study had an RNA integrity number value greater than 7. The recovered RNA was stored at – 80 °C until cDNA synthesis.

### cDNA synthesis

cDNA was generated using the SuperScript™ VILO™ cDNA Synthesis Kit (Invitrogen, Thermo Fisher Scientific, Carlsbad, USA) according to the manufacturer’s instructions. cDNA was aliquoted and stored at – 20 °C until used for qPCR.

### Quantitative PCR

Canine reference and target immune genes used in this study [23, 31, 32] are listed in Table 1. PCR amplification was performed using the QuantStudioTM 12K Flex System Real-Time PCR (Thermo Fisher Scientific) using TaqMan® Universal Master Mix II with UNG (Applied Biosystems, Foster City, USA). Plates (96-well plates) were filled with 0.35 µl nuclease free water (Sigma-Aldrich, San Luis, USA), 7.50 µl TaqMan Universal Master Mix (2×), 0.75 µl TaqMan assay 20 and 6.4 µl 1/5 cDNA. Plates were closed with an optical film (Applied Biosystems) centrifuged in order to mix the samples and placed into a laboratory pipetting robot (Epmotion 5057 Liquid-handlingrobot, Eppendorf, Hamburg, Germany) to generate a 384-well plate. Then, the generated 384 well plates were transferred into a real time PCR device. The PCR components and the PCR cycler conditions were identical for the all target and reference genes. Denaturation program (95 °C, 10 min), amplification and quantification program were repeated 40 times (95 °C for 15 s, 60 °C for 10 s, 72 °C for 60 s) with a single fluorescence measurement. The baseline and threshold were automatically defined for the program in each run. Each sample was performed in triplicate for all the target and reference genes and a calibrator sample (one sample from Group C) was employed as control in each plate. All target genes per each dog were run on the same day and in the same plate. Data were processed while applying the relative quantification method comparable to the delta-delta-quantification cycle value (ddCq) method. For normalization of target gene expression, the arithmetic mean of the two reference genes were taken for the calculation of a reference gene index [23]. Quantitative PCR data analyses was done by the Cloudsuite software (Life technologies^TM^, Thermo Fisher Scientific).

**Table 1:** Canine reference and target immune genes used in the present study

Assay ID^a^	Gene symbol	Gene name	GenBank mRNA	GenBank reference sequence	Amplicon size
Cf02625049_s1	TLR2	Toll-like receptor 2	AF328930.1	NM_001005264.2	69
Cf02622203 g1	TLR4	Toll-like receptor 4	AB080363.1	NM_001002950.1	120
Cf02710573_s1	TLR7	Toll-like receptor 7	AB248956.1	NM_001048124.1	124
Cf02624265_m1	IL-10	Interleukin 10	AF328930.1	NM_001003077.1	83
Cf02623316_m1	IFN-γ	Interferon gamma	AF091130.1	NM_001003174.1	117
APG2FND	PD-L1	Programmed dead ligand 1	NM_001291972.1	NM_001291972.1	164
Cf02643820_m1	LOC479750	Similar to CG14980-PB	XM_536878.2	XM_536878.2	78
Cf02664981_m1	SDHA	Succinate dehydrogenase complex; subunit A; flavoprotein	XM_535807.2	DQ402985.1	64

^a^All the assays are commercially available from Thermo Fisher Scientific

### Skin parasite load

DNA was purified from the interphase and organic phase generated from the RNA purification process by means of QIAamp DNA Mini Kit (Qiagen, Manchester, UK) following the manufacturerʼs instructions with slight modifications. Briefly, 20 μl of proteinase K solution and 200 μl of tissue sample were used in all samples. The other steps were performed as per manufacturerʼs protocol. A fragment of skin from a control dog was used as a control for DNA contamination during DNA extraction. qPCR was performed with *L. infantum* specific oligonucleotide primers N13A (5ʹ-AAC TTT TCT GGT CCT CCG GG-3ʹ) and N13B (5ʹ-CCC CCA GTT TCC CGC CC-3ʹ) were used to amplify a 120-bp fragment of the *Leishmania* kinetoplast DNA minicircle as previously described [7]. The parasite load was measured with the calculation of the delta Cq (dCq = mean values of duplicate determination of *Leishmania* Cq -*18S* rRNA Cq). Therefore, low or negative values of dCq represented higher parasite load than elevated dCq.

### Whole blood IFN-γ release assay

An IFN-γ release whole blood cultured assay was performed as described previously [33]. Briefly, 500 µl of heparinized whole blood was separately mixed with 4.5 ml of three different conditions: (i) unstimulated medium; (ii) medium with soluble *L. infantum* antigen (LSA, 5 mg/ml, Facultat de Farmacia, Universitat Autònoma de Barcelona) at a concentration of 10 µg/ml; and (iii) medium with the mitogen concanavalin A (ConA, 100 mg, Medicago, Uppsala, Sweden) at a concentration of 10 µg/ml. IFN-γ was determined in supernatants obtained five days after stimulation by a commercial sandwich ELISA (DuoSet ELISA by Development System R&D^TM^, Abingdon, UK). Cytokine concentration from supernatants with ConA and LSA was calculated after subtracting the IFN-γ concentration obtained from unstimulated supernatants.

### Statistical analysis

Statistical analysis was performed using the IBM SPSS Statistics software (version 1.0.0.1032) (SPSS Inc., Chicago, USA) and the *blorr*, *generalhoslem* and *Deducer* packages of the R software i386 version 3.4.2 (R Development Core Team) for Windows software. Categorical data were expressed as percentages and statistical analysis was performed using the Fisher’s exact test to compare independent variables. Quantitative data are expressed as the means ± standard deviation (SD). The non-parametric Wilcoxon signed-rank test and Mann-Whitney U-test were used to compare related and independent variables, respectively. The Spearman’s rank order correlation between transcripts in skin samples and immunological (*L. infantum* specific antibody levels and blood IFN-γ production), clinicopathological and parasitological data was also calculated.

Multivariable logistic regression was constructed to assess the relationships between all transcripts studied and skin *Leishmania* qPCR in clinically-lesioned skin when compared with normal-looking skin. The model was performed with *blorr*, *generalhoslem* and *Deducer* packages of the R software. Only quantitative and qualitative variables with a *P*-value of equal or less than 0.2 based on the univariate analysis were included in the model. Models were performed with data for all dogs and with data obtained from dogs of Group A and B, separately. The response variable was the type of skin (clinically lesioned or normal-looking skin) and the explanatory variables were the results of the skin *Leishmania* qPCR (numeric result and interpretation) and the transcripts (TLR2, TLR4, TLR7, IL-10, IFN-γ and PD-L1). Multivariate models were constructed in a stepwise fashion, beginning with a full model and removing variables one-by-one. Those variables included in the model were assessed for collinearity by Spearman’s correlation test, a result of ≥ 0.7 between two variables was assessed as two non-independent correlated variables and the variable with the weakest relationship with the response variable (higher *P*-value) was excluded. Goodness-of-fit was assessed by deviance of the residuals, ruling out overdispersion. A ROC curve was used to validate the model. Differences were considered significant with a 5% significance level (*P* < 0.05).

## Results

### Dogs

Eleven purebred dogs belonging to ten breeds and 14 mixed-breed dogs were included. Both sexes were represented by 11 females and 14 males. The median age was 2.5 years with a range from five months to 10 years. Specifically, dogs from Group A were six females and five males with a median age of 10 months, whereas dogs from Group B were five females and nine males with a median age of 54 months. Age difference was statistically significant among groups (Mann-Whitney U-test, *Z* = − 2.773, *P* = 0.006). All Beagle healthy dogs (Group C) were male and between three and six years of age.

### Histology

Histological features of the skin of diseased dogs (Group A and B) were previously described elsewhere [7]. Briefly, normal-looking skin of dogs from Group B was more frequently inflamed than normal-looking skin of dogs from Group A (78.6% and 27.3%, respectively; Fisher’s exact test, *P* = 0.017). The perivascular to interstitial inflammatory pattern was more common in clinically-lesioned skin from dogs from Group B than in clinically-lesioned skin from dogs from Group A (71.4% and 18.2%, respectively; Fisher’s exact test, *P* = 0.015). On the other hand, nodular to diffuse pattern with granuloma formation was more common in clinically-lesioned skin from dogs from Group A than in clinically-lesioned skin from dogs from Group B (36.4% and 0%, respectively; Fisher’s exact test, *P* = 0.017).

### Immunological, parasitological and clinicopathological data

Control dogs from a non-endemic area (Group C) were deemed clinically healthy seronegative non-infected dogs and were not included in comparisons reported in this section. The most relevant evaluated parameters studied in diseased dogs are listed in Table 2. As expected, dogs classified in Group A were in a less severe disease status than dogs classified in Group B as they had significantly lower values for total proteins, beta and gamma globulins and higher values for albumin/globulin ratio, hematocrit and hemoglobin. Moreover, dogs from Group B had significantly higher levels of specific antibodies and skin parasite load in dogs with both clinically-lesioned and normal-looking skin than in dogs from Group A.

**Table 2:** Clinicopathological data, antibody levels, IFN-γ production in stimulated blood and skin parasite load of sick dogs (groups A and B)

Parameter (reference intervals and units)	Group A (*n* = 11)	Group B (*n* = 14)	*P*-value	*Z*-value
UPC (< 0.5)	0.2 ± 0.1	0.7 ± 0.7		
Creatinine (0.5–1.5 mg/dl)	0.8 ± 0.1	0.9 ± 0.2		
Urea (21.4–59.9 mg/dl)	43 ± 14	31.3 ± 7.8	0.037	− 2.083
Total protein (5.4–7.1 g/dl)	5.7 ± 0.6	9.1 ± 2.4	0.0001	− 3.686
Albumin (2.6–3.3 g/dl)	3 ± 0.4	2.3 ± 0.8		
Beta globulin (0.9–1.6 g/dl)	1.3 ± 0.3	1.9 ± 0.6	0.01	− 2.572
Gamma globulin (0.3–0.8 g/dl)	0.7 ± 0.9	3 ± 2.6	0.002	− 3.079
Abumin/globulin ratio	1.1 ± 0.2	0.5 ± 0.4	0.004	− 2.899
Hematocrit (37–55 %)	45.6 ± 8.5	33 ± 9.8	0.024	− 2.251
Hemoglobin (12–18 g/dl)	15.4 ± 2.4	11.1 ± 3.8	0.032	− 2.148
*Leishmania infantum* specific antibody levels (cut-off value 35 EU units)	136.8 ± 196.1	8892.7 ± 17807.7	< 0.0001	− 3.887
Blood *L. infantum* specific IFN-γ (pg/ml)	2046 ± 2746.6	713.7 ± 832.8		
Blood parasite load (parasites/ml)	7.8 ± 10.5	6.7 ± 11.1		
Skin parasite load (dCq)				
Clinically-lesioned	3.6 ± 4.4	− 0.4 ± 4.7	0.043	− 2.026
Normal-looking	6.1 ± 4	1.7 ± 4.5	0.004	− 2.869

*Notes*: Low or negative values of dCq represent higher parasite load than elevated dCq. Differences were assessed *via* Mann–Whitney test

*Abbreviations*: UPC, urinary protein/creatinine ratio; EU, enzyme-linked immunosorbent assay units; dCq, delta-cycle threshold

### Transcripts in clinically-lesioned skin in comparison with healthy skin from control dogs

**Fig. 1:**
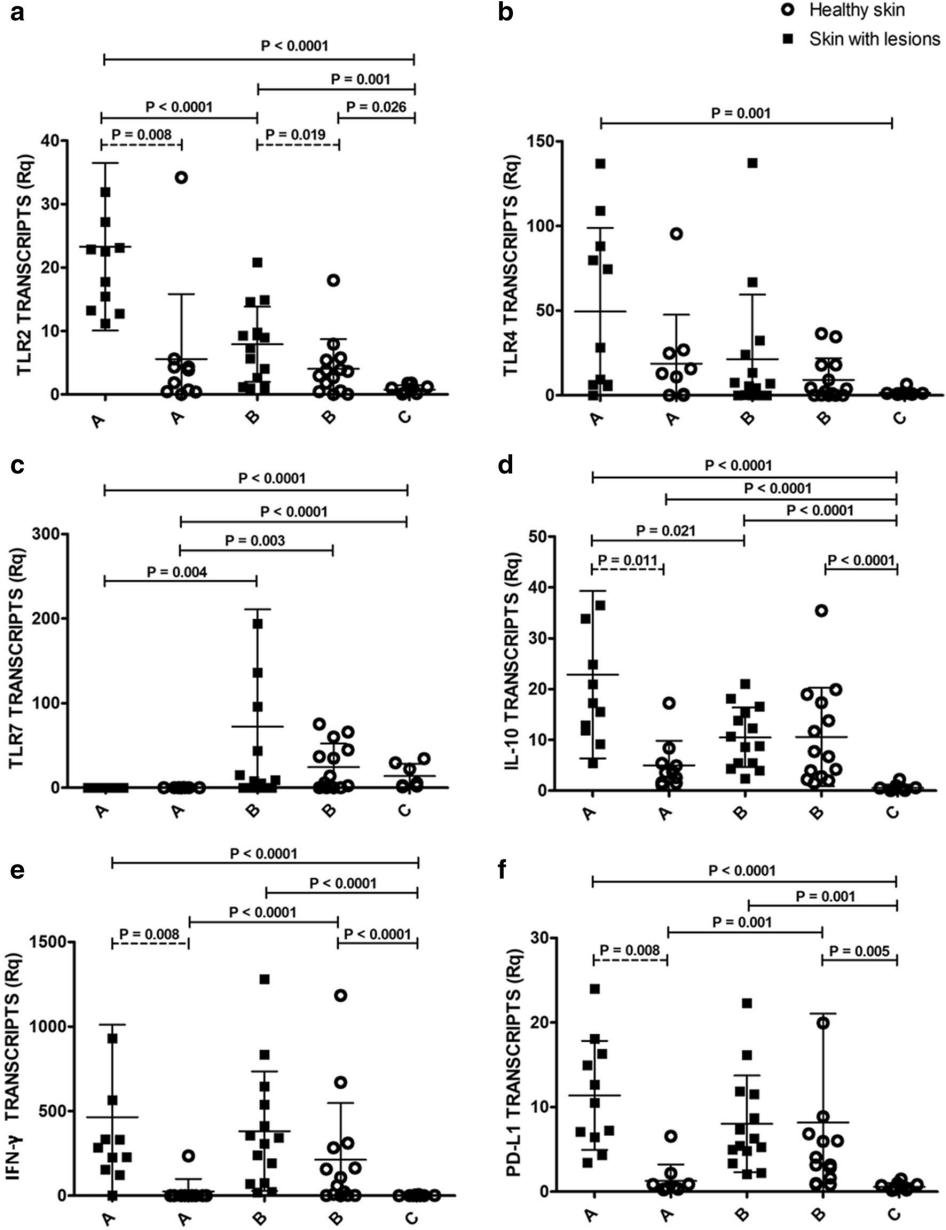
Relative quantification of the immune genes studied. **a** TLR2 transcripts. **b** TLR4 transcripts. **c** TLR7 transcripts. **d** IL-10 transcripts. **e** IFN-γ transcripts. **f** PD-L1 transcripts. Circles and squares represent individual data of each dog. Horizontal and vertical lines represent mean and standard deviation, respectively. Solid lines with *P*-values: Mann-Whitney U-test; dashed lines with *P*-values: Wilcoxon signed-rank test. *Abbreviations*: A, clinically-lesioned skin and normal-looking skin from Group A (stage I-mildly affected dogs); B, skin from Group B (stage II-III-severely affected dogs); C, skin from Group C (healthy non-infected dogs). Rq, normalized relative quantification

Relative quantifications of the expression of the immune response genes analyzed in the present study are shown in Fig 1. All transcripts except TLR7 were significantly increased in clinically-lesioned skin from dogs of Group A when compared with Group C (Fig. 1). In Group A, TLR7 was significantly downregulated. On the other hand, although all the transcripts were higher in clinically-lesioned skin from dogs of Group B when compared with Group C, only TLR2, IFN-γ, IL-10 and PD-L1 were significantly upregulated (Fig. 1).

### Transcripts in clinically-lesioned skin in comparison with paired normal-looking skin from sick dogs

Dogs from Group A showed significant upregulation of TLR2, IL-10, IFN-γ and PD-L1 in clinically-lesioned skin when compared with paired normal-looking skin. In contrast, in Group B only TLR2 transcript was significantly higher in clinically-lesioned skin when compared with paired normal-looking skin (Fig. 1).

### Transcripts in clinically-lesioned skin in sick dogs with different clinical staging

Clinically-lesioned skin from dogs of Group A showed significant upregulation of TLR2 and IL-10 and downregulation of TLR7 in comparison with clinically-lesioned skin from dogs of Group B (Fig. 1). Although non-statistically significant, a trend for an upregulation of TLR4 and IFN-γ was also observed in Group A.

### Transcripts in normal-looking skin from sick dogs in comparison with healthy skin from control dogs

Relative quantification of TLR7 and IL-10 was significantly downregulated and upregulated, respectively, in the skin from dogs of Group A when compared with the skin from dogs of Group C (Fig. 1). Although all the transcripts were higher in normal-looking skin from dogs of Group B when compared with Group C, only TLR2, IFN-γ, IL-10 and PD-L1 were significantly upregulated.

### Transcripts in normal-looking skin in sick dogs with different clinical staging

Normal-looking skin from dogs of Group A showed significant downregulation of TLR7, IFN-γ and PD-L1 in comparison with normal-looking skin from dogs of Group B (Fig. 1). Although non-statistically significant, a trend for a downregulation of TLR2 and IL-10 was also observed in Group A when compared with Group B.

### Correlations with transcripts in clinically-lesioned skin and clinicopathological, immunological and parasitological findings

**Fig. 2:**
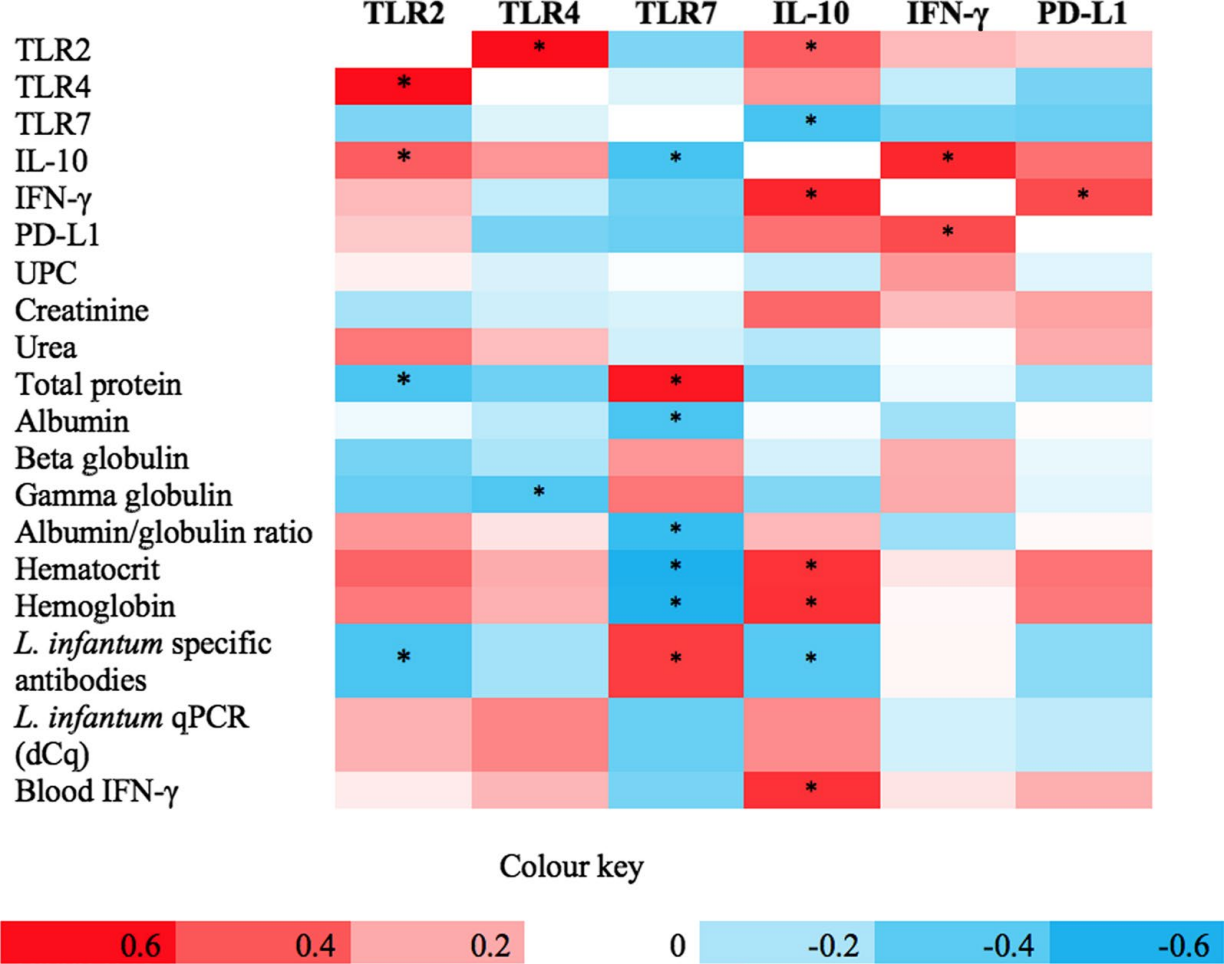
Heatmap illustrating the positive (red) and negative (blue) correlations between immune gene transcripts in clinically-lesioned skin from Group A (stage I- mildly affected dogs) and Group B (stage II-III-severely affected dogs) and clinicopathological, immunological and parasitological findings (correlations with *P* < 0.05 are indicated by an asterisk)

Correlations between transcripts in clinically-lesioned skin from sick dogs and the different parameters are illustrated in the heatmap shown in Fig 2. A significant positive correlation was noted between TLR2, TLR4 and IL-10 transcripts, whereas a significant negative correlation was observed between TLR2 and total protein and specific *L. infantum* antibodies. TLR4 in addition was negatively correlated with gamma globulin concentration. TLR7 was the transcript with more significant correlations obtained. In fact, it was associated with clinicopathological parameters suggestive of disease severity. Furthermore, IL-10 was positively correlated to skin IFN-γ transcript, hematocrit, hemoglobin and blood IFN-γ production, whereas a negative correlation between IL-10 and specific antibodies was observed. Skin IFN-γ transcript, in addition to the aforementioned correlations, was positively correlated with PD-L1.

### Correlations with transcripts in normal-looking skin and clinicopathological, immunological and parasitological findings

**Fig. 3:**
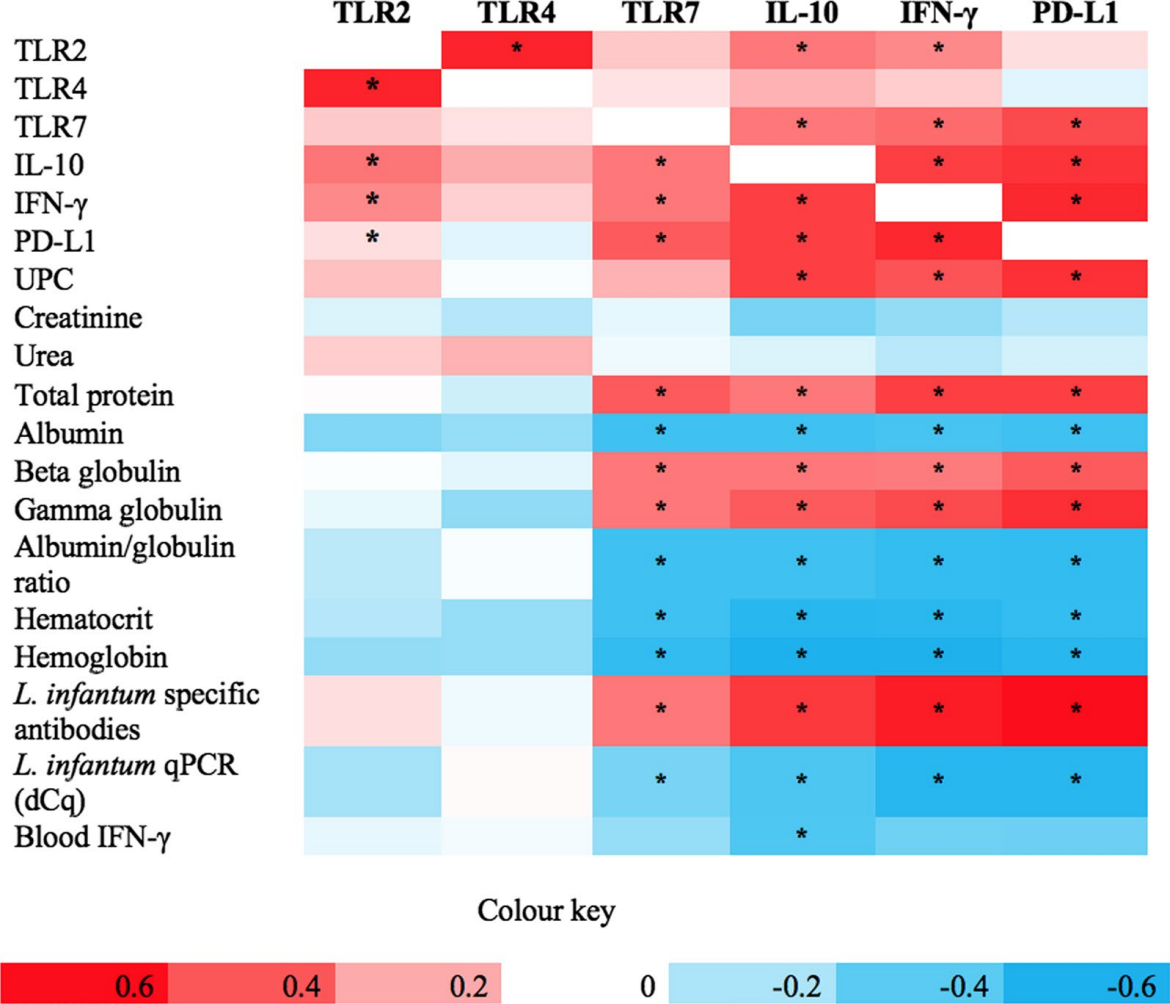
Heatmap illustrating the positive (red) and negative (blue) correlation values between immune gene transcripts in normal-looking skin from Group A (stage I-mildly affected dogs) and Group B (stage II-III-severely affected dogs) and clinicopathological, immunological and parasitological findings (correlations with *P* < 0.05 are indicated by an asterisk)

More significant correlations were determined between transcripts and clinical, immunological and parasitological findings in normal-looking skin than in clinically-lesioned skin (Fig. 3). The TLR2 transcript was positively correlated with TLR4, IL-10 and IFN-γ. A significant positive correlation was obtained between TLR7 and IL-10, IFN-γ, PD-L1, total protein, beta and gamma globulins and specific *L. infantum* antibodies. TLR7 was negatively correlated with albumin, albumin/globulin ratio, hematocrit, hemoglobin, and *Leishmania* dCq in qPCR. IL-10 showed similar correlations than TLR7, except for a positive correlation with TLR2 and UPC ratio and a negative correlation with blood IFN-γ production. Skin IFN-γ was positively correlated with TLR2, TLR7, IL-10, PD-L1, UPC ratio, total protein, beta and gamma globulins and specific *L. infantum* antibodies. In addition, there was a negative correlation between skin IFN-γ and albumin, albumin/globulin ratio, hematocrit, hemoglobin and *Leishmania* dCq in qPCR. Finally, PD-L1 was positively correlated with TLR7, IL-10, IFN-γ, UPC ratio, total protein, beta and gamma globulins and antibody levels, whereas a negative correlation was observed between this transcript and albumin, albumin/globulin ratio, hematocrit, hemoglobin and *Leishmania* dCq in qPCR.

A low dCq means higher skin parasite load. Therefore, negative correlations determined between dCq values and TLR7, IL-10, IFN-γ and PD-L1 mean an association between an upregulation of these transcripts and a high parasite load in normal-looking skin.

### Multivariable logistic regression models

A multivariable logistic regression model was constructed with data of all dogs. The model showed that TLR2 and IFN-γ transcripts are upregulated in clinically-lesioned skin from dogs with CanL (odds ratio > 1). The model developed with data of Group A did not show any statistical significance, whereas for Group B showed TLR2 upregulation (odds ratio > 1). The results are summarized in Table 3.

**Table 3:** Multiple logistic regression models performed with all data available that presented statistical significance

	Variables	Coefficient (β)	SE	*χ*^2^	*P*-value	Odds ratio	95% CI
All dogs (clinically-lesioned *vs* normal-looking skin) (*n* = 35)	Intercept	− 1.99	0.52				
	TLR2 transcript	0.240	0.060	6.790	0.009*	1.29	0.62–2.73
	IFN-γ transcript	0.003	0.001	4.570	0.033*	1.07	0.52–2.23
Group B (clinically-lesioned *vs* normal-looking skin) (*n* = 14)	Intercept	− 1.150	0.910				
	TLR2 transcript	0.300	0.150	4.120	0.042*	1.34	0.47–3.82
	IL-10 transcript	− 0.260	0.160	2.790	0.095	0.78	0.26–2.15
	IFN-γ transcript	0.003	0.002	3.230	0.072	1.00	0.35–2.85
	PD-L1 transcript	0.160	0.110	2.190	0.139	1.16	0.40–3.29

*Notes*: Coefficient is the mathematical weightings of the explanatory variables in the equation. Intercept is a mathematical constant of the model (no clinical interpretation)

*Abbreviations*: CI, confidence interval; SE, standard error; group B, stage II-III-severely affected dogs

*P < 0.05

## Discussion

This study aimed to investigate, to our knowledge for the first time, the transcription of TLR2, TLR4, TLR7, IFN-γ, IL-10 and PD-L1 in paired clinically-lesioned and normal-looking skin from the same dogs with different clinical stages and degrees of disease severity. More significant differences among immune gene transcripts in normal-looking skin and clinically-lesioned skin were determined in mildly affected dogs than in more severely affected dogs. This was not surprising considering that normal-looking skin of more severely affected dogs present an increased frequency of microscopic inflammatory lesions and higher parasite load than in normal-looking skin of mildly affected dogs [7].

TLR2 was significantly upregulated in clinically-lesioned skin of sick dogs when compared with healthy skin of non-infected dogs as previously documented in other tissues including the intestine [19], brain [20, 24], peripheral lymphoid organs [16, 20], liver [16], blood [23] and skin [16, 17], and this was associated with disease severity and progression. Although a lower expression of TLR2 in clinically-lesioned skin of dogs with papular dermatitis than in the skin of more severely affected dogs was initially suggested [25], the present results contradict the results of [25]. This discrepancy might be related to the retrospective design of the study by Esteve et al. [25], the lower number of cases included and because TLR2 expression has been measured by means of immunohistochemistry, a technique that is less accurate and sensitive than qPCR. Moreover, the discrepancies might be because the two techniques are evaluating different parameters (protein *versus* mRNA expression). Similar to the present study, TLR2 gene expression has been documented in different clinical presentations of tegumentary leishmaniasis in humans [34]. Mild forms of the disease (i.e. localized cutaneous leishmaniasis and borderline disseminated cutaneous leishmaniasis) caused by *L. braziliensis* has been reported to present higher TLR2 expression than the severe form mucosal leishmaniasis [34]. In addition, TLR2 transcript was negatively correlated with total protein and specific antibody levels in clinically-lesioned skin, rendering our finding, those dogs with stage I presented higher TLR2 transcript in clinically-lesioned skin, reasonable. TLR2 might induce an adequate proinflammatory response to control *L. infantum* infection in the skin of dogs as previously suggested in humans [34].

Upregulation of TLR2 gene was evident in normal-looking skin of dogs naturally affected by leishmaniosis [17]. In the present study, only more severely affected dogs showed a significant overexpression of TLR2 in normal-looking skin when compared with healthy skin of non-infected dogs, and, although not statistically significant, dogs with papular dermatitis showed lower TLR2 gene expression than more severely diseased dogs. This difference may be related to the increased frequency of microscopic inflammatory lesions and higher parasite load in normal-looking skin of more severely than in mildly affected dogs [7]. Taken all these findings together, it would seem that in more severely affected dogs there is a progressive TLR2 downregulation from earlier stages of inflammation to more chronic dermatitis. This reflection agrees with the observation of Hosein et al. [16] who described an upregulation of TLR2 in the skin only in the earlier stages of an experimental infection when compared with the controls [16].

TLR4 has been scarcely studied up to now in CanL in several tissues [16, 20, 23, 24], but, to the best of our knowledge, never in lesioned skin of dogs with leishmaniosis. Most of previously published data indicate an upregulation of TLR4 in several tissues such as spleen, lymph nodes [20] or brain [24] from sick dogs. In the present study the TLR4 transcript was significantly increased in clinically-lesioned skin of dogs with papular dermatitis compared with control skin. In addition, a higher TLR4 transcription level was observed in these dogs than in more severely affected dogs, although the difference was not significant. Moreover, a negative correlation among this transcript and gamma globulins is, to our knowledge, demonstrated for the first time, suggesting an association with less disease severity. Also, an organ compartmentalization of TLR4 gene expression could be possible as no differences were found in TLR4 relative quantification in non-stimulated blood between mildly and more severely affected dogs [35]. Nonetheless, and similar to TLR2, milder forms of human cutaneous leishmaniasis due to *L. braziliensis* are associated with higher expression of TLR4 [34]. Moreover, TLR4 polymorphisms have been associated with susceptibility to cutaneous leishmaniasis in humans [36, 37]. Therefore, TLR4 might induce an adequate proinflammatory response to control *L. infantum* infection in the skin of dogs as previously suggested in humans [34].

There are limited studies that determine TLR7 transcripts in CanL and TLR7 transcription appears to be unchanged in brain and spleen [24] as well as in canine monocyte-derived macrophages [22]. This TLR has been rarely studied in canine skin [38] and it has never been studied in the skin of dogs infected with *L. infantum*. Interestingly, in the present study, TLR7 gene expression was significantly lower in dogs with both clinically-lesioned and normal-looking skin with papular dermatitis than in more severely diseased dogs. Moreover, TLR7 overexpression in either in clinically-lesioned and normal-looking skin was associated with altered clinicopathological parameters suggestive of disease severity. Based on these results, a pathogenic role of this innate receptor in CanL is likely. In fact, recent evidence associated TLR7 activation with disease exacerbation of visceral leishmaniasis due to *L. donovani* in mice [39, 40]. Endosomal TLR7 activation in B cells by *L. donovani* has been suggested to be responsible for disease exacerbation through IL-10 and IFN-type I production and for the promotion of hypergammaglobulinemia [39]. Moreover, local tissue damage mediated by persistent inflammation has been reported to lead to suppression of protective T cell responses during chronic visceral leishmaniosis due to *L. donovani* in mice *via* signaling of TLR7 by apoptotic cell material [40].

Cytokine studies on clinically-lesioned skin are very limited [14]. Noteworthy, in the present study, IL-10 gene expression was studied in clinically-lesioned skin, to our knowledge, for the first time. A significantly higher IL-10 gene expression in papular dermatitis than in the skin lesions of more severely affected dogs was found and a positive relationship of this cytokine expression with parameters associated to disease control was observed. IL-10 is an immunoregulatory cytokine with multiple roles in immunopathology [41] but the roles of IL-10 in CanL remain uncertain. However, it seems that IL-10 is not a marker of disease severity at least in clinically-lesioned skin as previously observed in IFN-γ whole blood release assays (WBA) [13, 33] opposite to reports in mice and humans [42, 43]. In contrast, polysymptomatic-diseased, naturally infected dogs have presented an increased IL-10 production by T lymphocytes from blood along with increased blood parasite burden [44]. As previously described [13, 15, 17] and in agreement with the present findings, an upregulation of IL-10 in normal-looking skin of dogs with leishmaniosis was detected and associated with parameters of disease severity such as parasite density. In summary, higher levels of IL-10 gene expression would be an immunological parameter marker of disease severity in normal-looking skin but not in clinically-lesioned skin.

As expected, an upregulation of the IFN-γ transcript was observed in clinically-lesioned skin from dogs with leishmaniosis, both in mildly and more severely affected dogs, when compared with healthy skin of non-infected dogs. This result agrees with those previously published [14]. Although not statistically significant, a higher IFN-γ gene expression was observed in clinically-lesioned skin of mildly affected dogs when compared with more severely affected dogs. IFN-γ is a protective Th-1 associated cytokine, which increases the leishmanicidal activity of macrophages [2, 11]. Therefore, it is plausible that overexpression of this pro-inflammatory cytokine in mildly affected cases may be the result of granuloma formation in papular lesions with consequent lower parasite density as previously demonstrated [7, 25].

The relative levels of IFN-γ in normal-looking skin from mildly affected dogs were significantly lower than in normal-looking skin from more diseased dogs and were associated with disease severity (high specific antibody levels and high parasite density) [13]. The lower inflammation observed microscopically in normal-looking skin from mildly affected dogs may account for this finding [7]. This result is in line with the results of a previous study on normal-looking skin from naturally infected dogs demonstrating increased IFN-γ expression in symptomatic dogs in comparison with asymptomatic dogs [15]. Therefore, this pro-inflammatory environment observed in normal-looking skin of more severely affected dogs is not enough to confer protection, as previously suggested [13].

To the best of our knowledge, PD-L1 expression has never been investigated in the skin of dogs with leishmaniosis. An increase of PD-L1 in clinically-lesioned and normal-looking skin of dogs with leishmaniosis was demonstrated. Therefore, this overexpression may suggest a role of PD-L1 in the immunopathogenesis of CanL. This protein is related to a decreased T-cell mediated immunity due to T-cell exhaustion *via* its union with PD-1 on T-cells surface [26]. As suggested in human leishmaniasis, expression of PD-L1 might represent a mechanism that parasites exploit to avoid the host immune response [45]. However, similar expression in clinically-lesioned skin was observed among different disease stages. This was an unexpected finding as higher T-cell apoptosis was hypothesized in clinically-lesioned skin of more severely affected dogs. It would be interesting to evaluate if further increase of the number of studied dogs would change this finding. On the other hand, it is possible that factors other than PD-L1 exist as a cause of suppression of Th1 cell effector function as previously suggested [46, 47].

A lower PD-L1 gene expression was determined in normal-looking skin from dogs with papular dermatitis than in more severely affected dogs in agreement with the lower inflammatory process observed in normal-looking skin of mildly affected dogs [7]. In accordance, positive correlations of PD-L1 transcript with clinicopathological parameters associated with disease severity, antibody levels and parasite density were detected in normal-looking skin. Therefore, PD-L1 is suggested as an immunological marker for disease severity only in normal-looking skin.

## Conclusions

This study demonstrated, to our knowledge for the first time, different expression profiles of immune genes in clinically-lesioned and normal-looking skin from dogs with leishmaniosis. Moreover, differences among mildly and more severely affected dogs were revealed. Clinically-lesioned skin from mildly affected dogs was characterized by a significant upregulation of TLR2 and IL-10 and downregulation of TLR7 when compared with skin from more severely affected dogs. On the other hand, normal-looking skin of mildly affected dogs was characterized by a downregulation of TLR7, IFN-γ and PD-L1 when compared with skin from more severely affected dogs. Therefore, these immunological conditions might favor the maintenance and replication of the parasite in the skin of more severely affected dogs leading to disease progression.

## Data Availability

Data supporting the conclusions of this article are provided within the article. The datasets used and/or analysed during the present study are available from the corresponding author upon reasonable request.

## Abbreviations

CD4: cluster of differentiation 4
CanL: canine leishmaniosis
ConA: concanavalin A
Cq: quantification cycle
DNA: deoxyribonucleic acid
ELISA: enzyme-linked immunosorbent assay
EU: ELISA units
IFN-γ: interferon-gamma
IL-10: interleukin-10
LSA: *Leishmania infantum* soluble antigen
qPCR: quantitative polymerase chain reaction
PD-L1: programmed cell death protein ligand 1
PRR: pattern recognition receptor
RNA: ribonucleic acid
Th1: type 1 T helper cells
Th2: type 2 T helper cells
TLR: toll like receptor
